# Supplementary figures and images for: Evolution of the nonsense-mediated decay pathway is associated with decreased cytolytic immune infiltration
Source: PLoS Comput Biol. 2019 Oct 28;15(10):e1007467. doi: 10.1371/journal.pcbi.1007467 (PMC6837539; doi:10.1371/journal.pcbi.1007467)

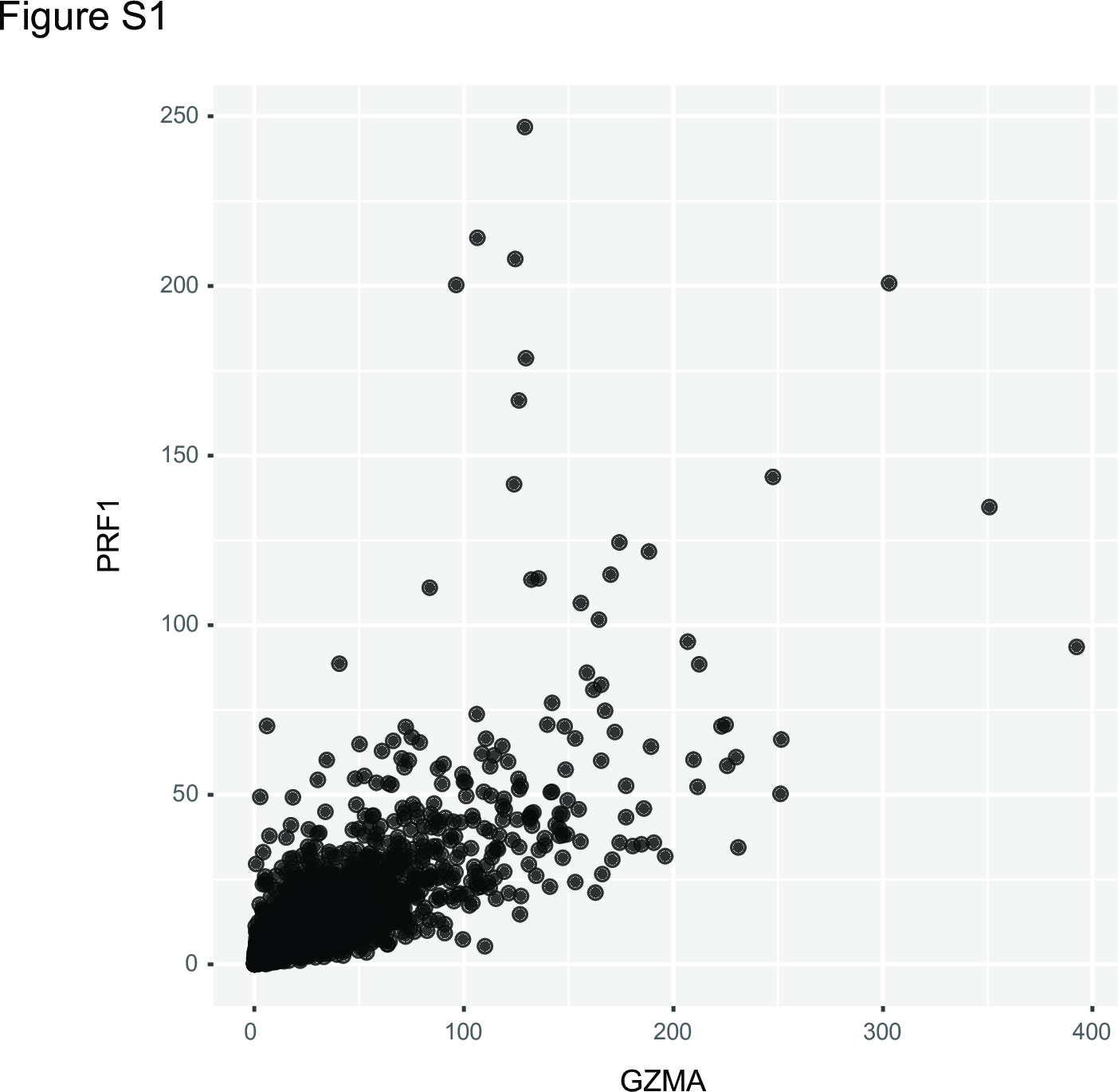

Supplement: S1 Fig — (A) Pan-cancer ROC curve for predicting cytolytic activity, using a random forest model with only counts of each mutation variant type (B) Out-of-bag error of overall model (black) and for predicting cytolytic activity low (red) and high (green). (C) Variable importance of the features used in the model, based on mean decrease in model accuracy. (D) Association in mutation counts among different mutation variant types. Missense, silent, and nonsense are correlated while frameshift is not. (TIF) [file pcbi.1007467.s003.tif]

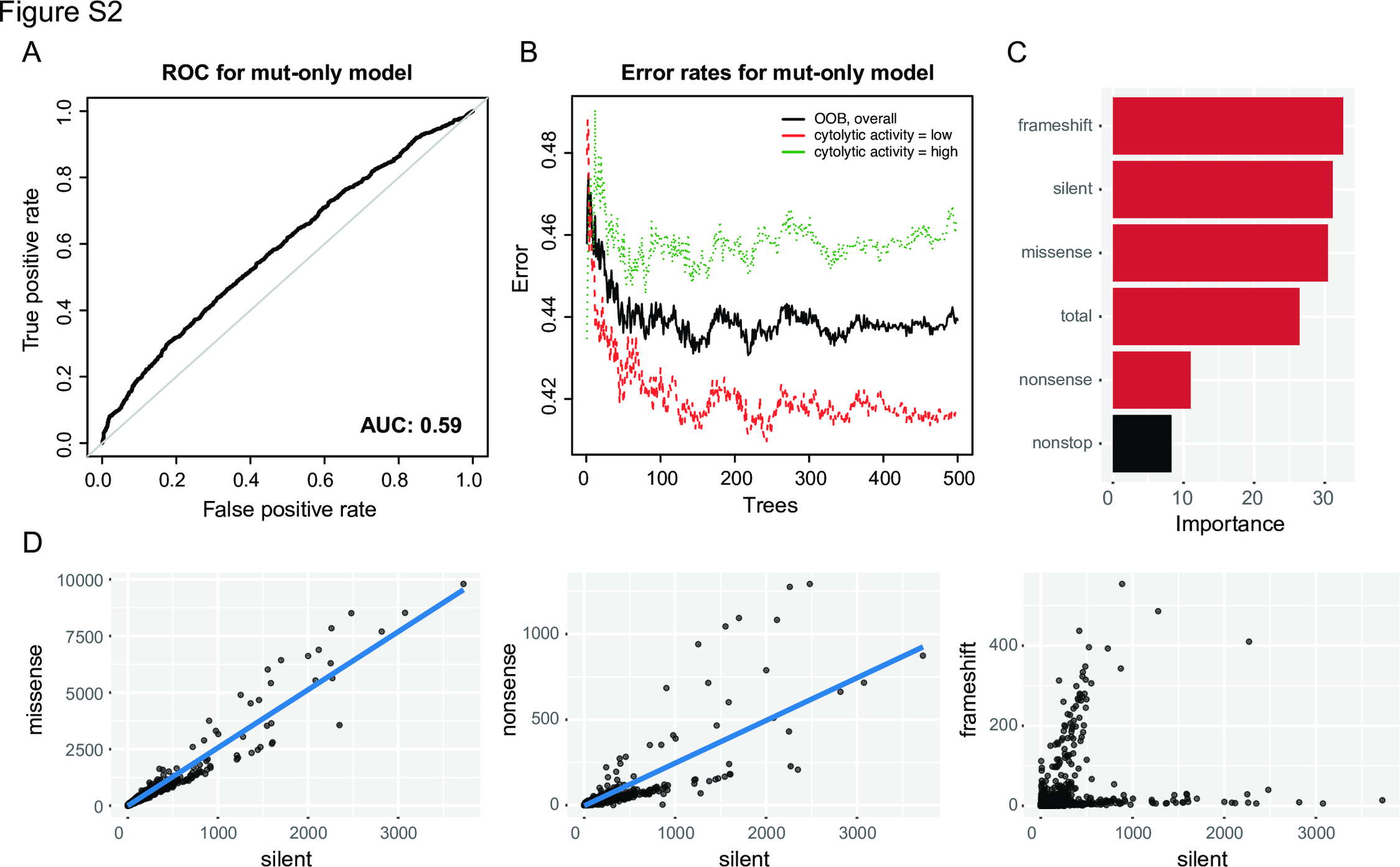

Supplement: S2 Fig — (TIF) [file pcbi.1007467.s004.tif]

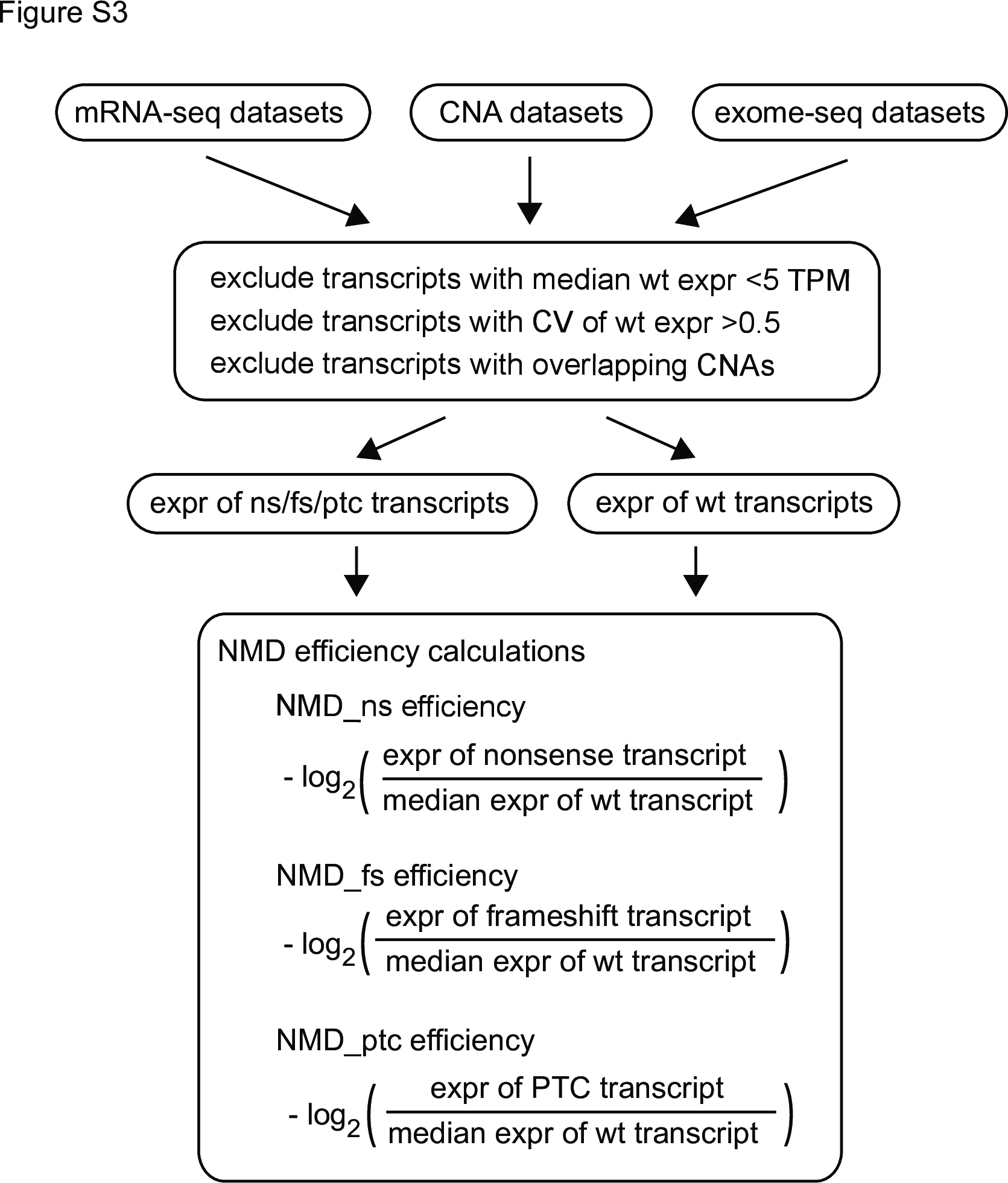

Supplement: S3 Fig — The mRNA-seq, CNA, and exome-seq datasets were incorporated. Noisy genes were filtered out, followed by derivation of gene-level NMD efficiency values. expr, expression. (TIF) [file pcbi.1007467.s005.tif]

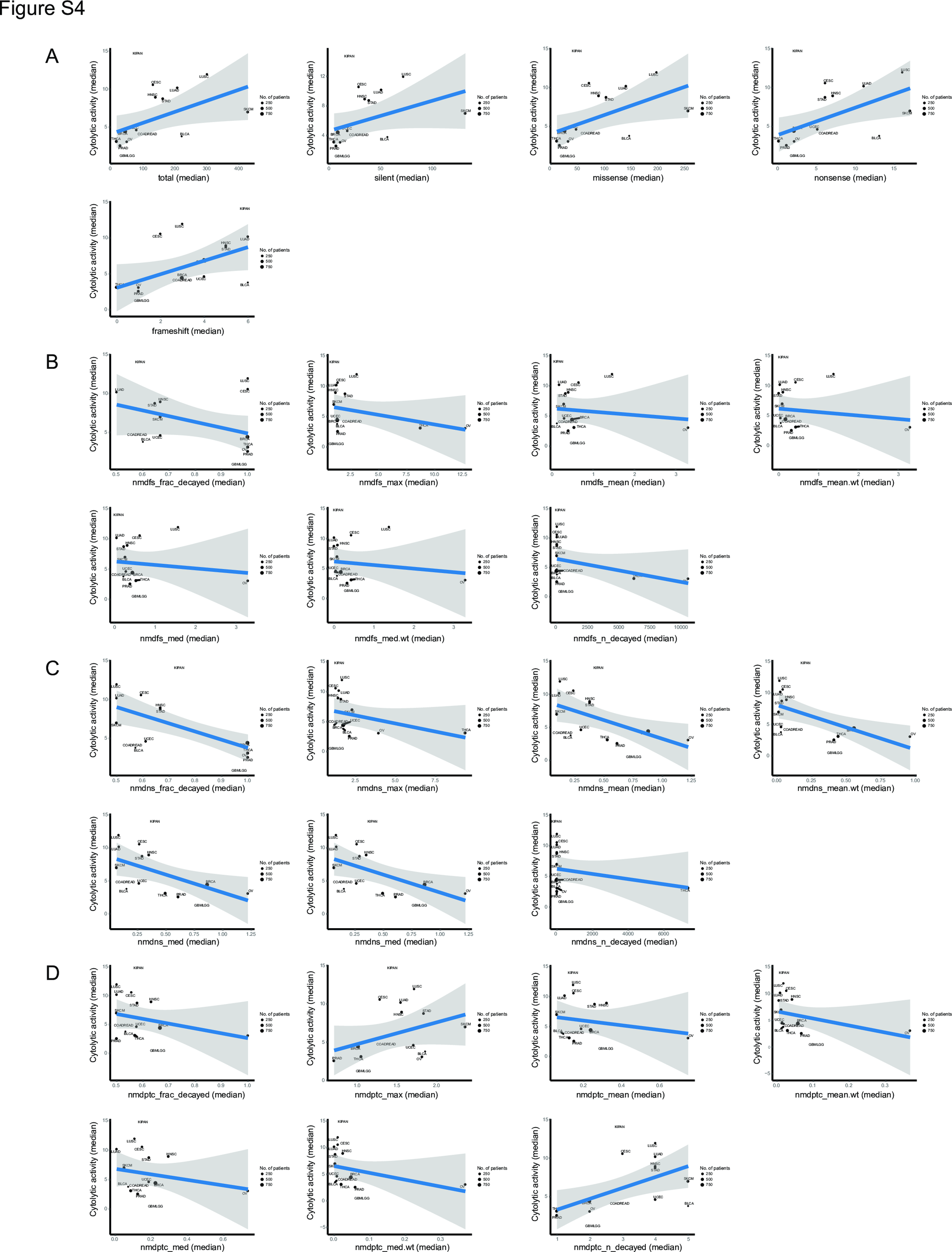

Supplement: S4 Fig — Features were grouped into mutations (A), NMD frameshift-bearing (fs) (B), NMD nonsense-bearing (ns) (C), and NMD nonsense/frameshift-bearing (ptc) (D). (TIF) [file pcbi.1007467.s006.tif]

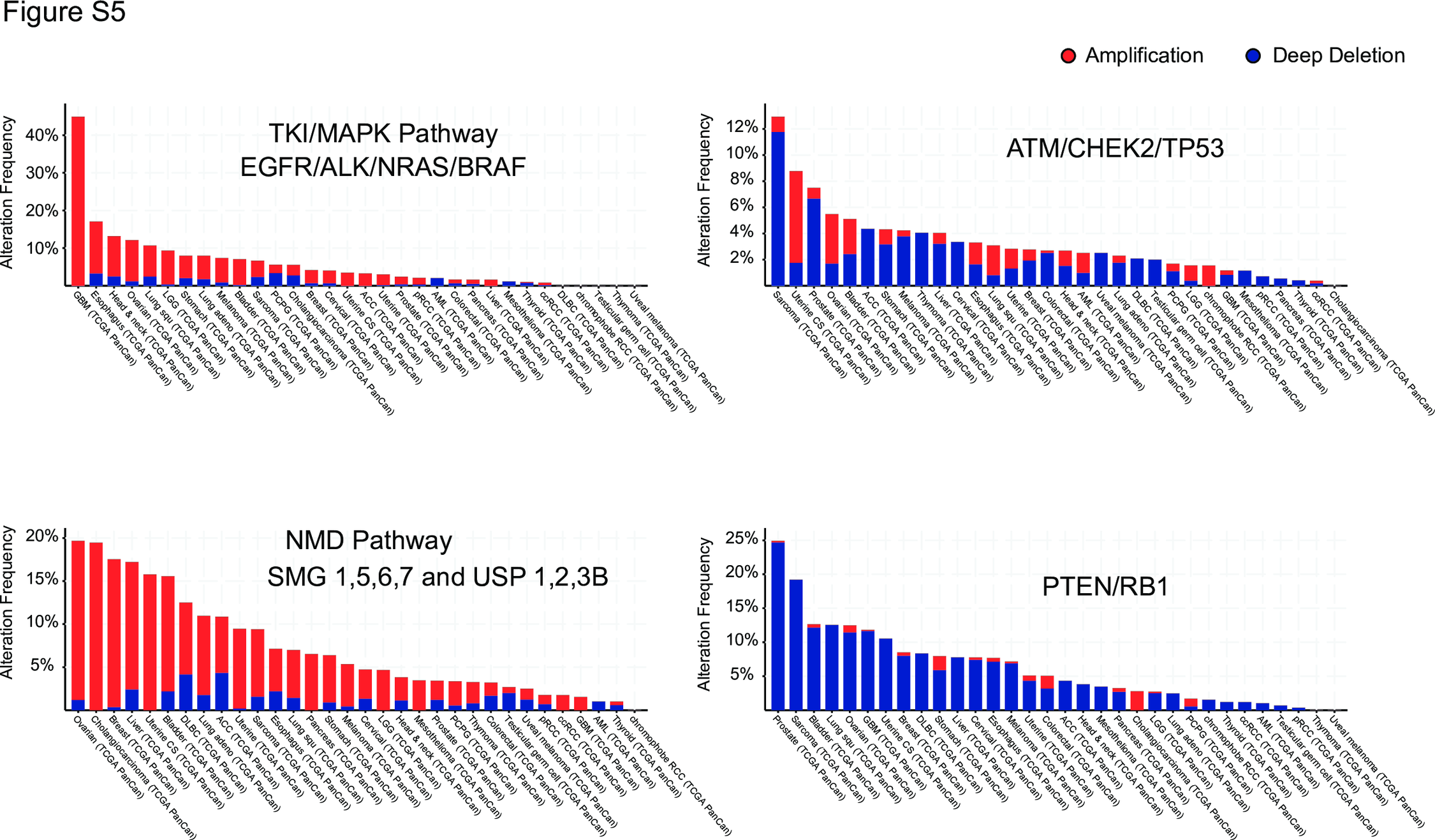

Supplement: S5 Fig — Amplifications are shown in red and deletions in blue. (TIF) [file pcbi.1007467.s007.tif]

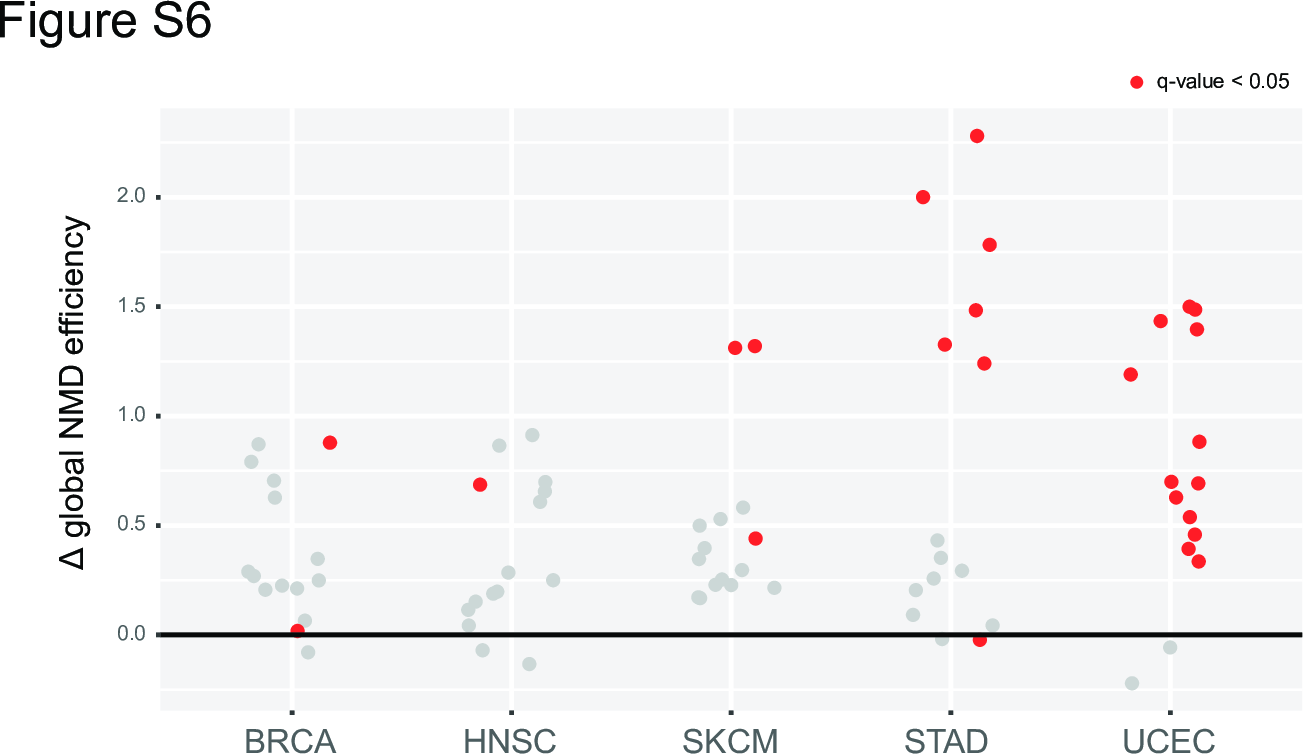

Supplement: S6 Fig — Y-values are shown as the difference in median of log10 transformed NMD metric values (co-altered versus no alterations). Dots shown in red are statistically significant with adjusted p-value < 0.05; Mann-Whitney test with Benjamini-Hochberg multiple hypothesis correction. (TIF) [file pcbi.1007467.s008.tif]

Figure S7

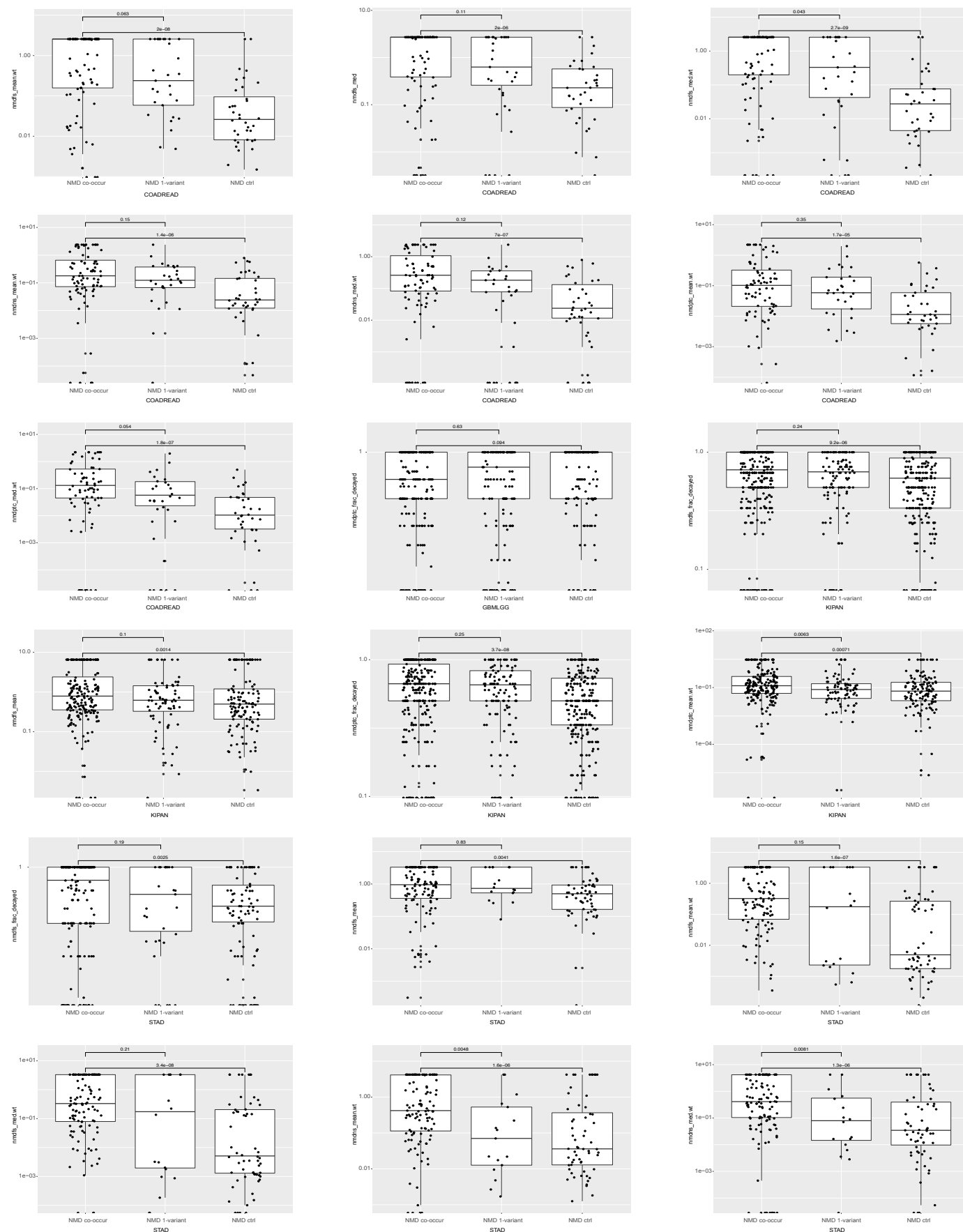

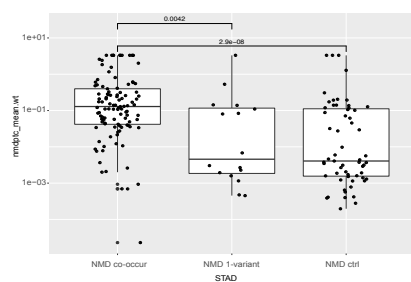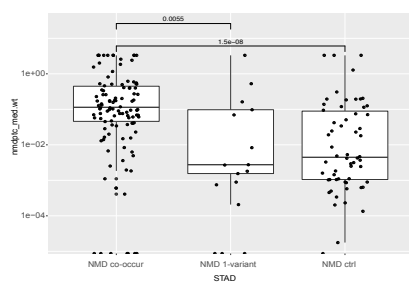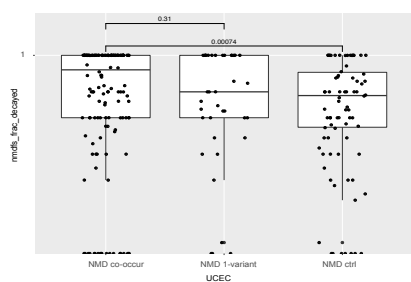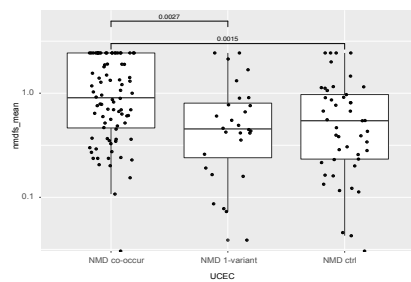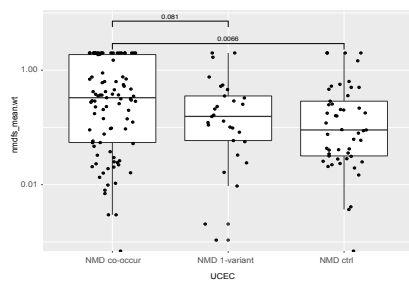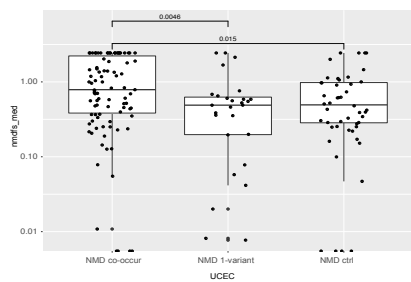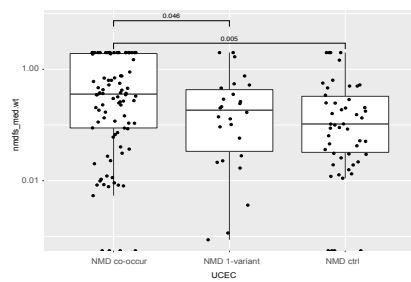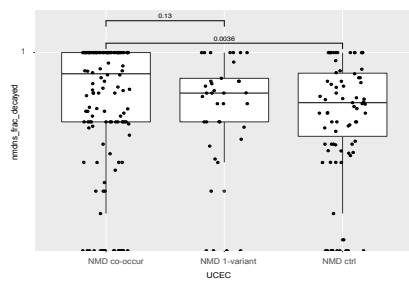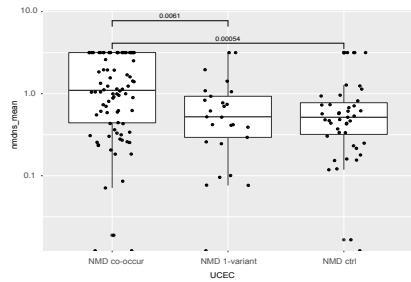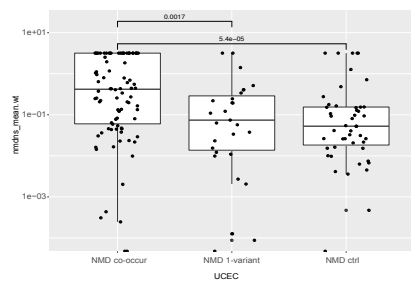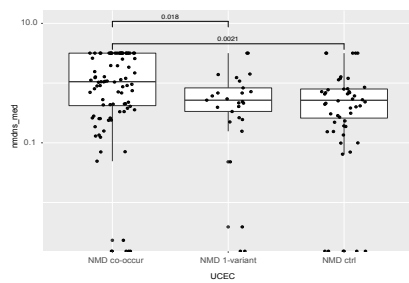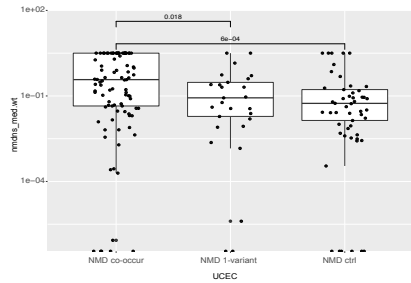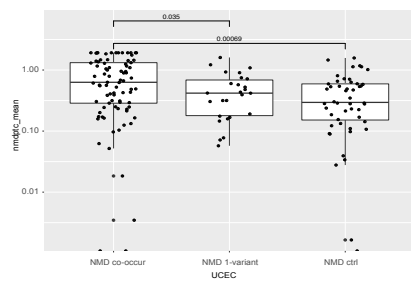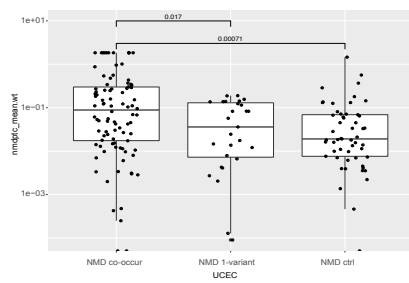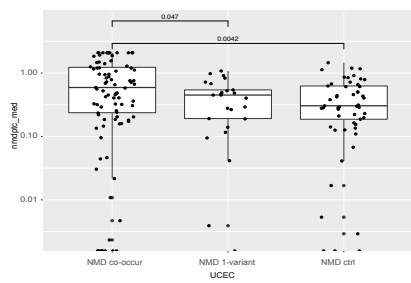

Supplement: S7 Fig — Statistical significance was assessed with Jonckheere-Terpstrata test for trends and p-values were multiple hypothesis corrected using the Benjamini-Hochberg method. Results with adjusted p-value < 0.05 are shown. The p-values shown in the plots are based on Mann-Whitney test of pair-wise comparisons. (PDF) [file pcbi.1007467.s009.pdf]

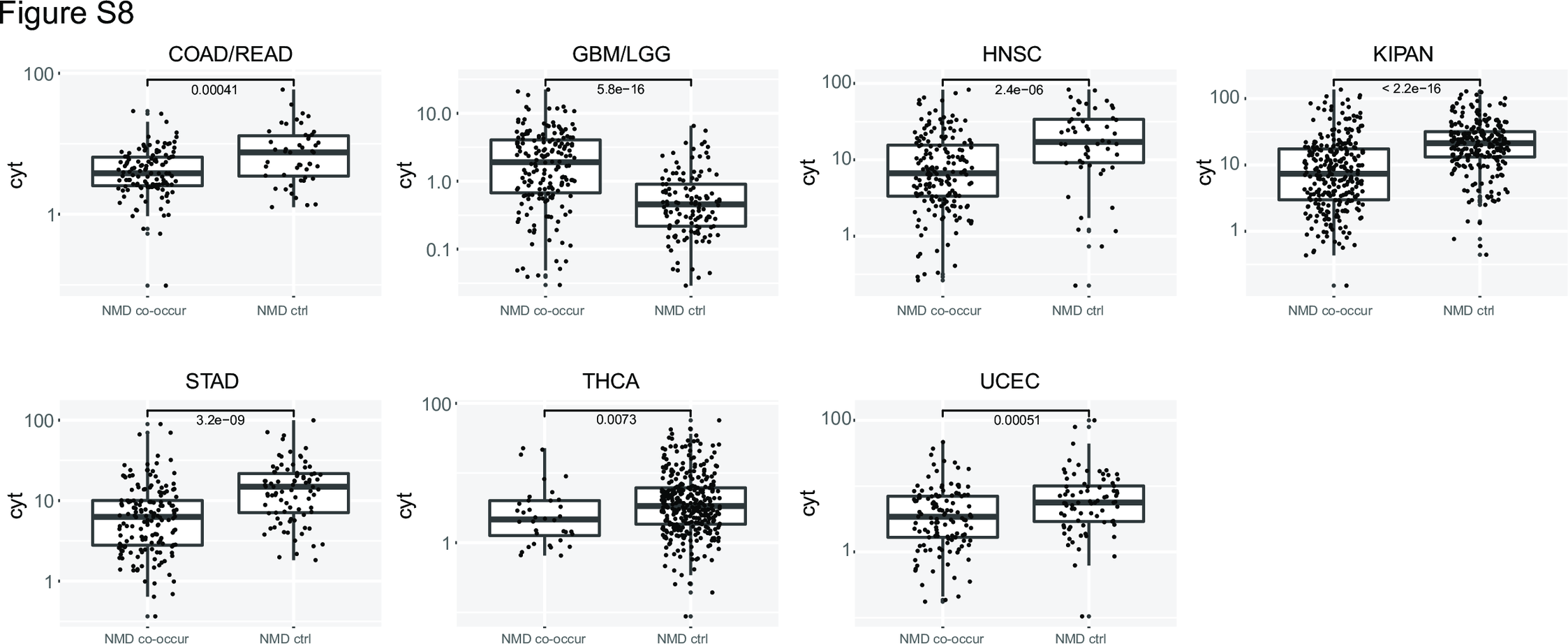

Supplement: S8 Fig — Statistical significance was assessed with Mann-Whitney test and p-values were multiple hypothesis corrected using the Benjamini-Hochberg method. Results with adjusted p-value < 0.05 are shown. Nominal p-values shown in plots. (TIF) [file pcbi.1007467.s010.tif]

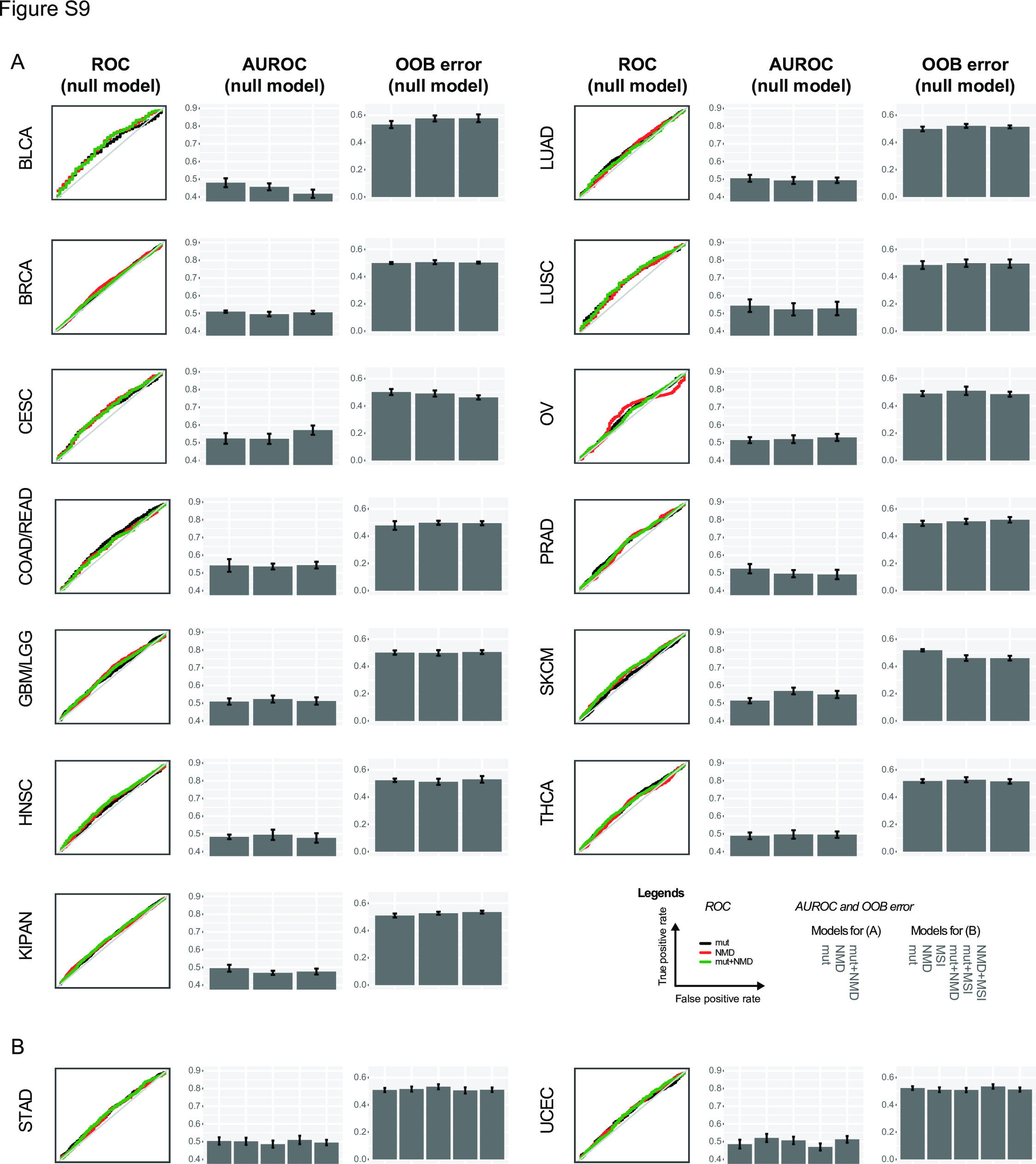

Supplement: S9 Fig — ROC, AUROC, and out-of-bag (OOB) error of random forest models, built from ten randomized datasets, for (A) indications without microsatellite instability (MSI), and (B) indications with MSI. AUROC data are shown as AUROC ± SE of AUROCs. (TIF) [file pcbi.1007467.s011.tif]

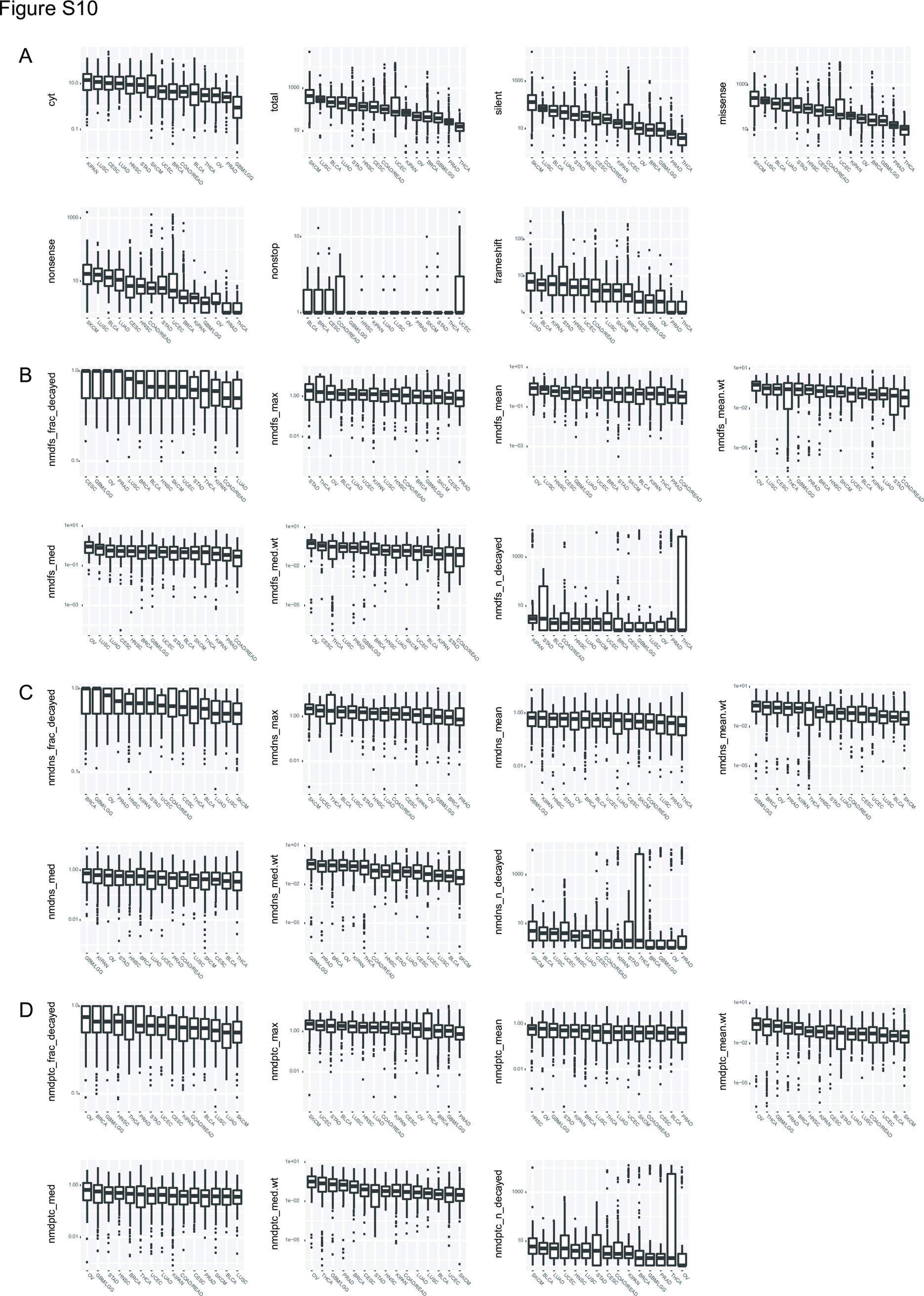

Supplement: S10 Fig — Features were grouped into mutations (A), NMD frameshift-bearing (fs) (B), NMD nonsense-bearing (ns) (C), and NMD nonsense/frameshift-bearing (ptc) (D). (TIF) [file pcbi.1007467.s012.tif]

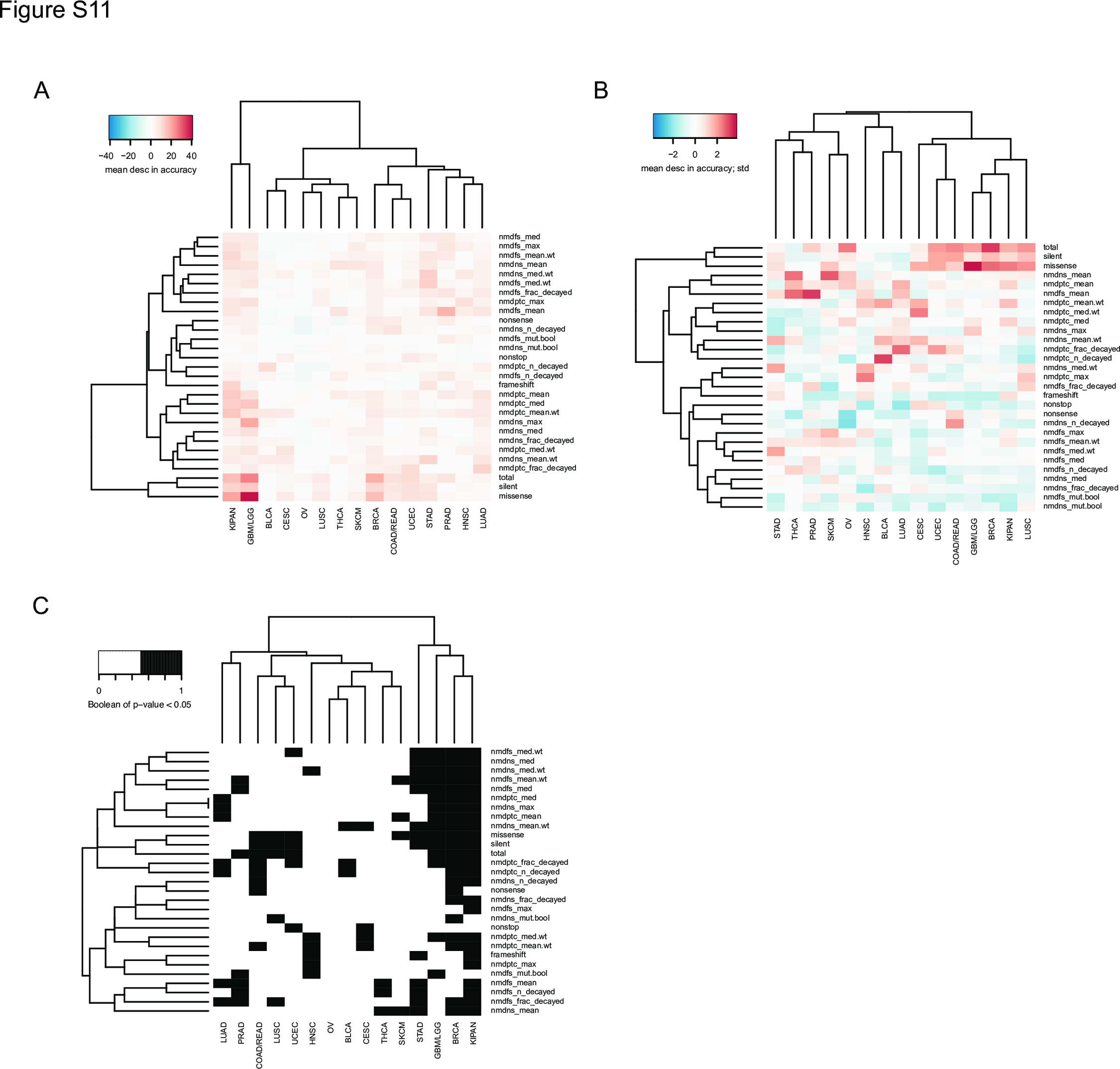

Supplement: S11 Fig — (A) Mean decrease in model accuracy when a given feature is removed from the model. (B) Standardized mean decease in accuracy values from (A). (C) Statistically significant features from the model, with p-value < 0.05 marked in black. (TIF) [file pcbi.1007467.s013.tif]

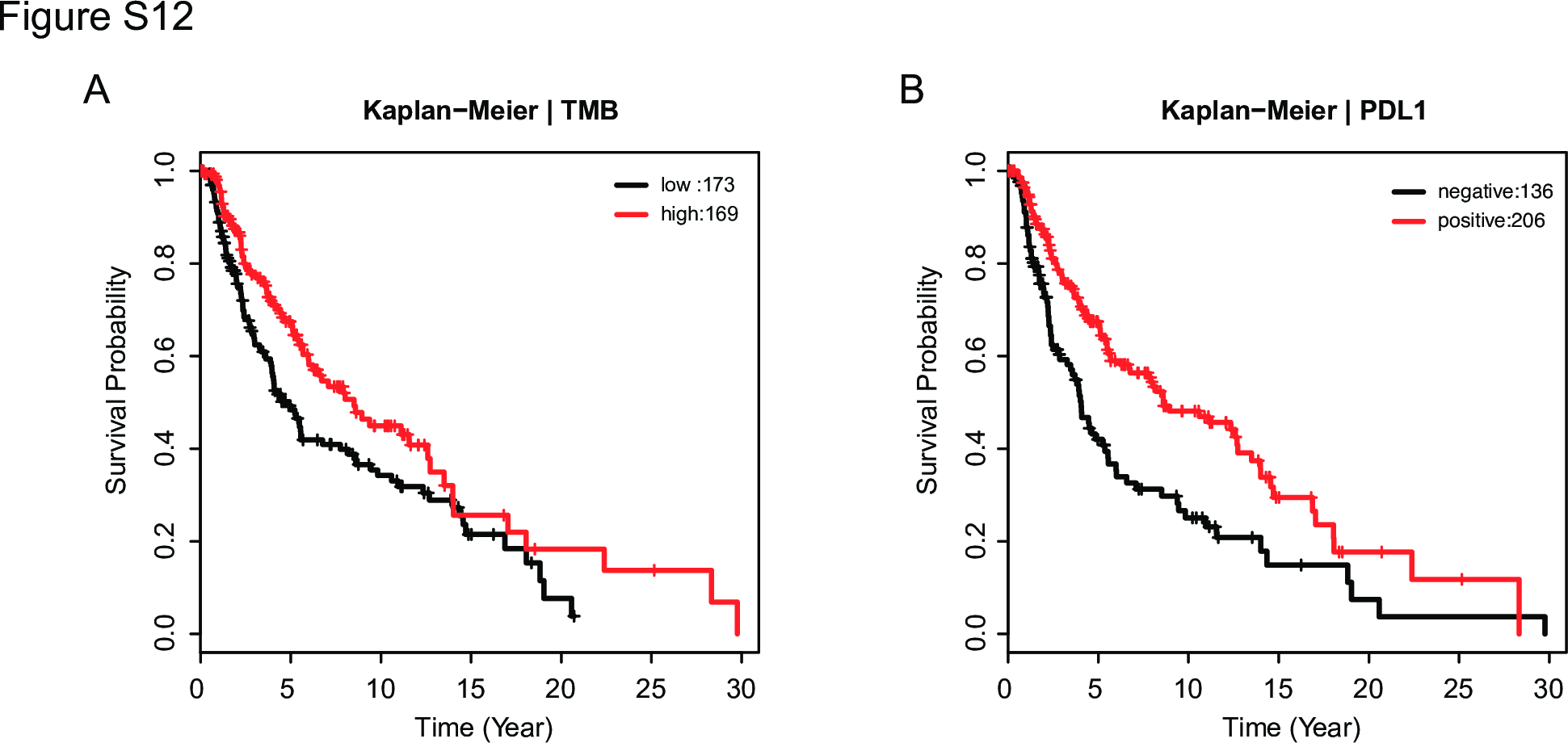

Supplement: S12 Fig — Univariate overall survival analysis of SKCM for TMB (A) and PDL1 (B). (TIF) [file pcbi.1007467.s014.tif]

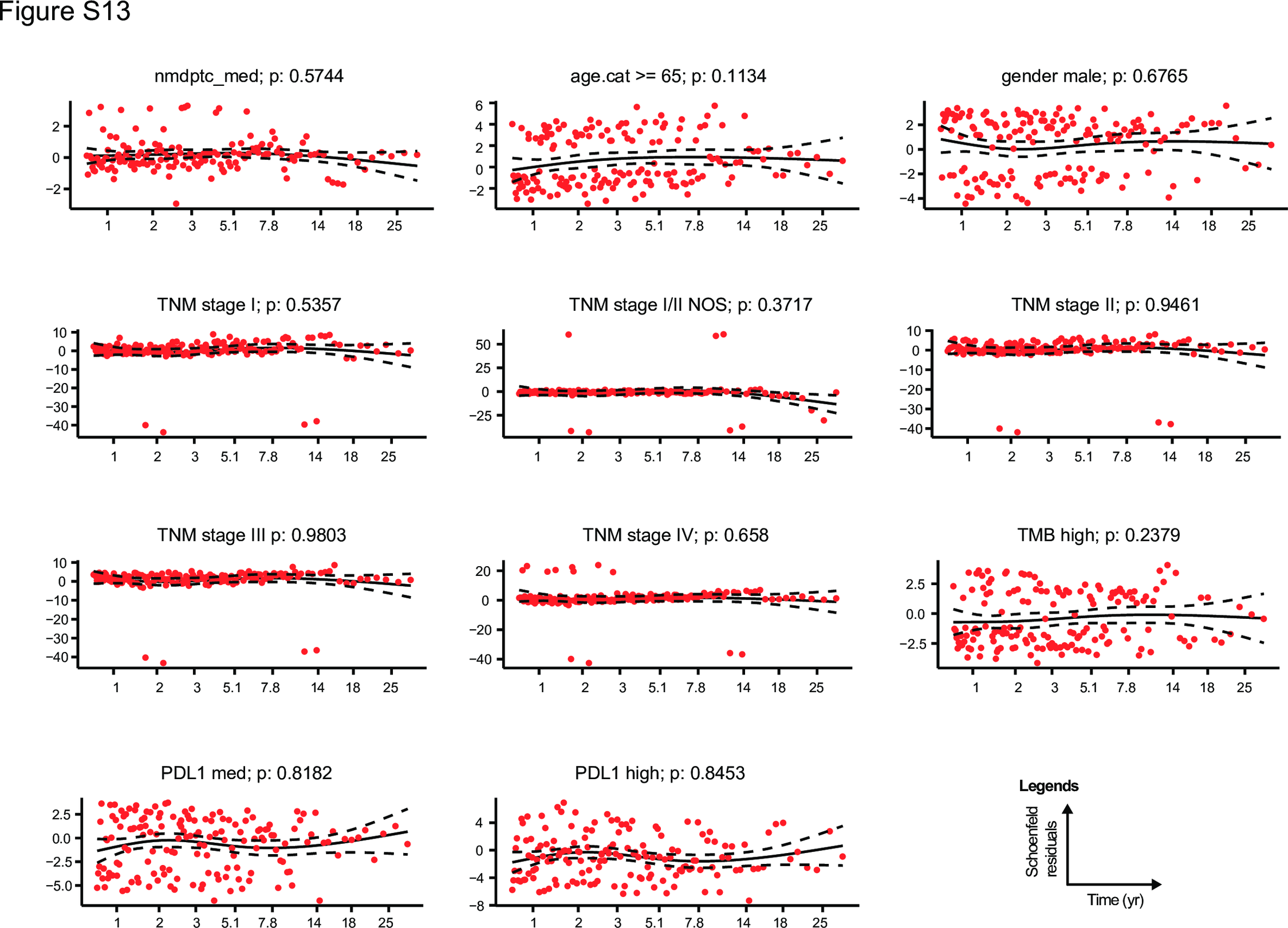

Supplement: S13 Fig — (TIF) [file pcbi.1007467.s015.tif]
